# Mobile Electroencephalography for Studying Neural Control of Human Locomotion

**DOI:** 10.3389/fnhum.2021.749017

**Published:** 2021-11-10

**Authors:** Seongmi Song, Andrew D. Nordin

**Affiliations:** ^1^Department of Health and Kinesiology, Texas A&M University, College Station, TX, United States; ^2^Department of Biomedical Engineering, Texas A&M University, College Station, TX, United States; ^3^Texas A&M Institute for Neuroscience, College Station, TX, United States

**Keywords:** EEG signal processing, motor neuroscience, neuroimaging, locomotion, mobile EEG, electroencephalography (EEG), EEG hardware

## Abstract

Walking or running in real-world environments requires dynamic multisensory processing within the brain. Studying supraspinal neural pathways during human locomotion provides opportunities to better understand complex neural circuity that may become compromised due to aging, neurological disorder, or disease. Knowledge gained from studies examining human electrical brain dynamics during gait can also lay foundations for developing locomotor neurotechnologies for rehabilitation or human performance. Technical barriers have largely prohibited neuroimaging during gait, but the portability and precise temporal resolution of non-invasive electroencephalography (EEG) have expanded human neuromotor research into increasingly dynamic tasks. In this narrative mini-review, we provide a (1) brief introduction and overview of modern neuroimaging technologies and then identify considerations for (2) mobile EEG hardware, (3) and data processing, (4) including technical challenges and possible solutions. Finally, we summarize (5) knowledge gained from human locomotor control studies that have used mobile EEG, and (6) discuss future directions for real-world neuroimaging research.

## Introduction

Understanding human brain processes during real-world behaviors is a major neuroscience challenge. Moving cognitive and motor neuroscience studies beyond stationary, seated experiments and into complex, realistic environments is a necessary step forward to decipher real-world human brain dynamics. Because walking is a fundamental motor task that can have profound effects on quality of life and requires complex interactions throughout the nervous system, there is a need to better understand healthy human neuromotor control and to identify pathways affected by a loss of neurological function due to disease, disorder, injury, or aging (Snijders et al., 2007). Although basic locomotor control can be primarily attributed to subcortical structures and spinal central pattern generators, a growing body of evidence has shown that cortical structures directly modulate locomotion, including motor planning, execution, and error correction. Understanding cortical involvement during locomotion is, therefore, necessary to improve clinical detection and rehabilitation results.

Contemporary brain imaging technologies can measure neural dynamics by capturing a number of contrasting physiological signals. Neural electromagnetic signals can be measured using electroencephalography (EEG) and magnetoencephalography (MEG), changes in blood oxygenation can be measured using hemodynamic measurement methods such as magnetic resonance imaging (MRI) and functional near-infrared spectroscopy (fNIRS), and molecular imaging methods such as positron emission tomography (PET) and single-photon emission computed tomography (SPECT) enable scientists and clinicians to non-invasively study human brain functions. Technological limitations, however, have largely limited neuroimaging studies to motionless conditions from participants who are seated or lying supine due to the physical size of the recording equipment or because of noise introduced by participant or equipment motions that compromise signal recording quality. In recent years, a growing need for continuous brain monitoring during movement has promoted the development of mobile brain/body imaging (MOBI) approaches. The advantages and limitations of each technology must be considered in the context of the temporal and spatial resolution of each system and the associated cost and portability. Although fNIRS can portably measure human brain hemodynamics, and advancements in MEG technologies that rely on novel optically pumped magnetometers (OPM) (Boto et al., 2018; Hill et al., 2020; Tierney et al., 2020) provide promising paths forward for studying real-world human brain dynamics, the low-cost portability and precise temporal resolution of EEG has enabled the expansion of human neuromotor research into more complex and dynamic tasks (Allali et al., 2018).

A primary limitation of mobile EEG for studying real-world human brain dynamics has been the need to eliminate noise contamination from scalp EEG recordings. During unconstrained movements such as walking, electrode motions on the scalp and cable sway increase along with electrophysiological signals from the heart, eye movements, and facial and neck muscle activities. Low signal-to-noise ratio and comparatively poor spatial resolution in relation to fMRI and molecular imaging methods have been progressively addressed through advanced signal processing to isolate and localize electrocortical source activity using independent component analysis and forward head modeling techniques (Delorme and Makeig, 2004; Acar and Makeig, 2010; Vorwerk et al., 2018). Leveraging these advancements, recent locomotion studies have extended our understanding of human brain activity during treadmill (Castermans et al., 2014; Nathan and Contreras-Vidal, 2016; Bradford et al., 2016; Nordin et al., 2019a) and overground locomotion (Luu et al., 2017a) in complex virtual (Luu et al., 2016, 2017b) and real-world environments (Bruijn et al., 2015; An et al., 2019; Peterson and Ferris, 2019), and during robotically assisted gait (Wagner et al., 2012; Li et al., 2018).

Improved capabilities for measuring real-world human brain dynamics using state-of-the-art mobile EEG technologies will continue to advance the field of human cognitive and motor neuroscience. Here, we identify current mobile EEG technologies and analysis methods that have enabled groundbreaking discoveries into human neuromotor control during locomotion, and we briefly summarize some of the remaining challenges and paths forward for human mobile brain and body imaging studies.

## Mobile EEG Hardware

Table 1 provides a summary of representative commercially available mobile high-density EEG system specifications. The range of electrode array density (number of channels), recording electrode type, system size, portability, and mass provide advantages and disadvantages for studying neural control of human locomotion. Here, we discuss considerations for measuring human electrical brain dynamics using contrasting mobile EEG system configurations.

**Table 1:** Mobile high-density EEG systems for studying neural control of human locomotion.

System	Channel density	Electrode type					Portability	Mass
		Active		Passive		Size (mm)		
		Wet	Dry	Wet	Dry			
**BIOSEMI**:ActiveTwo	Up to 271-channels (256 scalp+ 8ECG or EMG + 7 external channels)	√				120 × 150 × 190	Body worn recording hardware	1.1 kg
**Cognionics**:CGX MOBILE-128	Up to 128-channels (EEG)	√				90 × 60 × 20	Body worn recording hardware	1 kg (98 g:Recording system only)
**ANT Neuro**:eego sports	Up to 128-channels (EEG or EMG)			√	√	160 × 205 × 22	Body worn recording hardware	<500 g (Recording system only)
**G.Tec**:g. NAUTILUS RESEARCH HEADSET	Up to 64-channels (EEG)	√	√			78 × 60 × 26	Head worn entire system	<140 g (Recording system only)
**Brain Products**:LiveAmp 64	Up to 64-channels (EEG, EMG, ECG, EOG)	√	√	√		140 × 83 × 18	Body worn recording hardware	<130 g (Recording system only)
**EMOTIV**:EPOC flex	Up to 32-channels (EEG)			√		220 × 155 × 50	Head worn entire system	1 kg

### EEG Recording Electrodes

Non-invasive EEG signal acquisition occurs using electrodes placed on the scalp. The recorded signal represents the summation of post-synaptic electrical potentials from the underlying and surrounding brain structures (Teplan, 2002; Sanei and Chambers, 2013), together with electrical noise from the surrounding environment, recording equipment, and ongoing electrophysiological activity from the eyes (Dement and Kleitman, 1957; Overton and Shagass, 1969; Schlögl et al., 2007), heart (Stephenson and Gibbs, 1951; Park et al., 2002), muscles (Goncharova et al., 2003; Whitham et al., 2007; Muthukumaraswamy, 2013). Electroencephalographic signals measured from the scalp show microvolt-scale fluctuations (peak-peak range: 0.5 μV–100 μV; Teplan, 2002), while noise contamination can occur at the millivolt scale (1,000× greater amplitude). The electrode characteristics and scalp-electrode interface can have large implications on EEG signal quality. Typical EEG electrodes commonly use silver/silver chloride (Ag/AgCl) that interfaces indirectly with the scalp through a conductive gel in a so-called wet electrode configuration. The Ag/AgCl wet electrodes have favorable reliability and signal integrity, with a low electrical impedance that diminishes low-frequency noise compared to high impedance electrodes recording in a warm, humid environment (Kappenman and Luck, 2010; Laszlo et al., 2014; Mathewson et al., 2017; Hinrichs et al., 2020). Any EEG setup can cause discomfort for the participant during prolonged use, but the main drawback of wet EEG electrodes is that the conductive gel dries over time, which reduces signal recording quality.

Dry contact electrodes were proposed to resolve some of the disadvantages of wet electrodes (Taheri et al., 1994; Gargiulo et al., 2008; Lopez-Gordo et al., 2014) by enabling electrical brain recordings over longer durations without a need for conductive gel (Xu et al., 2017). Additional advantages of dry contact electrodes include reduced setup time and limited inconvenience to the participant. However, dry contact electrodes show a higher impedance range and are more vulnerable to motion artifacts than wet electrodes due to the sensitive direct skin-electrode interface that is critical to mobile EEG signal recording quality (Xu et al., 2017). To overcome this, pressure is often applied to the electrodes and scalp through mechanical tension in the setup, which can lead to discomfort.

Quasi-dry electrodes, which combine advantages from both wet and dry contact electrodes, have been introduced as an intermediate solution for robust EEG signal recording (Mota et al., 2013). The quasi-dry electrode has a hydrated local skin interface with a moisturizing solution drawn from a reservoir inside the electrode. The significant advantages of quasi-dry electrodes include maintenance of lower electrode impedance similar to wet electrodes, with reduced discomfort compared to dry electrodes (Xu and Zhong, 2018). Quasi-dry electrodes also allow long-term EEG measurements due to the small amount of moisturizing solution that spreads and dries on the scalp less than typical wet electrode conductive gel (Mota et al., 2013). Quasi-dry electrodes do, however, often require additional pressure placed on the electrode to dispense the gel, which can result in non-uniform scalp pressure and discomfort.

Novel electrode configurations have also been introduced, including concentric ring electrode designs (He et al., 2001; Besio et al., 2006, 2014). By simultaneously recording from multiple closely-spaced recording sites on each electrode, signal-to-noise ratio and EEG spatial resolution can improve compared to conventional recording electrodes. Tripolar concentric ring electrodes have even outperformed bipolar and quasi-bipolar electrode designs by calculating the surface Laplacian or spatial second derivative using a multi-point differential among concentric rings on each electrode (He et al., 2001; Besio et al., 2006, 2014). Flexible electrodes provide another promising opportunity for configuring mobile EEG systems by relying on compliant, lightweight materials that can improve user comfort and enable longer duration recordings (Wang et al., 2012; Debener et al., 2015; Someya and Amagai, 2019; Acar et al., 2019; Shustak et al., 2019). Non-invasive flexible EEG electrodes, such as tattoos (Kim et al., 2011; Shustak et al., 2019; Ferrari et al., 2020) and conductive textiles (Löfhede et al., 2010, 2012), are capable of measuring electrocortical signals from the scalp, but are compromised by hair underlying the recording surface (Löfhede et al., 2012; Casson, 2019) and have therefore typically been placed on the forehead or around the ears (Kim et al., 2011; Debener et al., 2015; Acar et al., 2019; Shustak et al., 2019). These approaches have remained limited in locomotion studies or restricted to motor-related brain regions (Bunge, 2004), but fully portable and wireless EEG recording hardware with soft scalp electrodes that minimally penetrate the epidermis can provide a viable alternative (Mahmood et al., 2021). Widespread adoption of these innovative technologies remains dependent on signal recording quality, the ability to record electrical brain activity across the entire scalp surface, and user comfort. Mobile EEG recording innovations will continue to emerge and improve our abilities for measuring robust electrocortical activity during human locomotion.

### EEG Signal Amplification

In addition to the skin-electrode interface, electrode configurations and system designs have been proposed to enhance EEG signal recording quality. One representative idea is to integrate miniature amplifiers on each electrode, in a so-called active electrode configuration. Because active electrodes amplify the EEG signal at the recording site, EEG signal quality can improve by minimizing noise induced by cable sway (Mathewson et al., 2017). The influence of signal pre-amplification on EEG data quality using active electrodes, however, likely depends on the overall system configuration and relative placement of the system components (Scanlon et al., 2020). Active electrode designs can also enable skin-electrode impedance monitoring periodically throughout a data recording session to ensure low scalp-electrode impedance and high signal quality are preserved over time (Patki et al., 2012). Compared to standard passive EEG electrode configurations, each active electrode amplifier is electrically powered, often requiring additional wiring (Xu and Zhong, 2018).

## Mobile EEG Data Processing

### Source Separation Methods for Artifact Removal

Measured at the scalp, EEG electrodes record electrical potentials from the brain that are a mixture of multiple source components (Hyvärinen and Oja, 2000). Blind source separation methods, specifically independent component analysis (ICA; Bell and Sejnowski, 1995; Makeig et al., 1997), can effectively decompose the channel-based electrode recordings into independent source components. Widely available through MATLAB-based open-source scripts in EEGLab (Delorme and Makeig, 2004), ICA is central to many mobile EEG studies for isolating electrocortical signals from the complex mixture of signal and noise measured from EEG channel recordings. Independent components that are isolated from the electrode channel recordings can then be categorized into brain components and other components such as noise or other physiological signals. Although numerous ICA-based algorithms exist for deriving source components from high-density EEG channel data, an adaptive mixture independent component analysis algorithm (AMICA; Palmer et al., 2012) that relies on an unsupervised learning approach has been reported to be most effective at reducing mutual information among ICA-derived source components (Delorme et al., 2012). This approach is also able to detect time-varying brain states through multiple modeling (Hsu et al., 2018), though sufficient data are needed from long-duration EEG recordings to effectively separate source signal components, which comes at a higher computational cost.

Distinguishing independent components that originate from the brain and non-brain sources is a critical step when studying human electrical brain dynamics during locomotion. In practice, identification criteria have included, but are not limited to, scalp topography, source dipole location, time series, and power spectrum. Subjective methods based on visual inspection and objective statistical criteria have each been used for selecting electrocortical source activity derived from ICA, but the effectiveness of these approaches is dependent on ICA decomposition quality and between-subject variability (Ullsperger and Debener, 2010). To increase consistency and efficiency for classifying brain and non-brain independent components, automatic classification data processing toolboxes available in EEGLab have been made available. Some of these toolboxes include ICLabel (Pion-Tonachini et al., 2019), MARA (Multiple Artifact Rejection Algorithm; Haresign et al., 2021), FASTER (Fully Automated Statistical Thresholding for EEG Artifact Rejection; Nolan et al., 2010), SASICA (Semi-Automated Selection of Independent Components of the electroencephalogram for Artifact correction; Chaumon et al., 2015), ADJUST (Automatic EEG artifact Detection based on the Joint Use of Spatial and Temporal features; Mognon et al., 2011), and IC_MARC (Frølich et al., 2015). Although these approaches can help to distinguish the brain and non-brain source components from ICA decomposition, visual inspection is still typically advisable.

Multivariate source separation techniques used in Brain-Computer Interface (BCI) applications, may also provide viable alternatives to ICA signal decomposition. Because BCI studies have fewer trials for real-time control, preprocessing is essential (Wolpaw and McFarland, 2004; Kübler et al., 2005; Blankertz et al., 2007), with data-driven supervised decomposition algorithms typically used as a spatial filter (Blankertz et al., 2007; Nikulin et al., 2011; Dähne et al., 2014; Haufe et al., 2014). Common Spatial Patterns (CSP; Müller-Gerking et al., 1999; Ramoser et al., 2000; Blankertz et al., 2007) generate spatial filters to improve BCI classification and can improve EEG signal quality by optimizing spatial filters based on predominant event-related desynchronization or synchronization (ERD: spectral power decrease and ERS: spectral power increase, respectively) within a certain frequency band compared between conditions (Blankertz et al., 2007). Source Power Comodulation (SPoC; Dähne et al., 2014) is designed to find spatial filters for extracting oscillatory signals from continuous variables, and when applied to scalp patterns from simulation data, improved ground truth source power estimation compared to ICA (Dähne et al., 2014). Spatio-spectral decomposition (SSD; Nikulin et al., 2011) has also been used to improve signal quality within specific frequency bands by estimating noise around the frequency range of interest. Because SSD assumes that noise spans a broad frequency range from a few Hz to tens of Hz, rather than white or 1/f noise (Nikulin et al., 2011), researchers should also consider noise traits specific to each dataset.

### Electrocortical Source Localization

Estimating the source locations of electrical brain activity using independent components derived from scalp EEG recordings requires the solution of a so-called inverse problem. That is, determine the source signal locations required to produce the mixture of signals recorded at the scalp electrodes. It is essential for clinical and functional brain research applications to identify the brain structures involved in a task or behavior (Cuffin, 1998; Keil et al., 2014), but finding accurate spatial source locations is difficult due to the effects of volume conduction (Jung et al., 2001) as the electrical source activity propagates through cortical tissues, cerebrospinal fluid, resistive scalp layers, and the skull (Burle et al., 2015). To solve the EEG inverse problem, various techniques, such as non-parametric and parametric methods, were proposed (Grech et al., 2008). Modeled as an electrical dipole, non-parametric approaches assume that source components distributed in the whole brain maintain fixed orientations. Such methods include LORETA (Low resolution electrical tomography; Baillet, 1998), VARETA (Variable resolution electromagnetic tomography; Valdes-Sosa et al., 2000; Bosch-Bayard et al., 2001), S-MAP (Spatial regularization; Baillet, 1998; Grech et al., 2008), ST-MAP (Spatio-temporal regularization; Baillet and Garnero, 1997; Grech et al., 2008), LAURA (Local autoregressive average; de Peralta Menendez et al., 2004), SSLOFO (Standardized shrinking LORETA-FOCUSS; Liu et al., 2005), and ALF (Adaptive standardized LORETA/FOCUSS; Schimpf et al., 2005). In contrast, parametric approaches consider dipole changes in time and try to search for the best dipole positions and orientations. These methods include FINES (First Principle Vectors; Xu et al., 2004), simulated annealing (Miga et al., 2002), and computational intelligence algorithms [e.g., Neural network (Robert et al., 2002) and Artificial neural network (Van Hoey et al., 2000)]. Source localization technologies exist to improve source location estimation by co-registering the precise location of EEG electrodes on the scalp with the subject-specific head anatomy. Imaging technologies, such as 3D scanning, ultrasound, optoelectronic, or camera-based computer vision methods, therefore, provide opportunities to improve electrical source localization accuracy (Koessler et al., 2010; Baysal and Sengül, 2010; Shirazi and Huang, 2019) when combined with forward head models that incorporate subject-specific MRI scans and conductivity estimates for anatomical head structures.

### Temporal and Spectral Dynamics

The millisecond temporal resolution of EEG enables the study of precise electrocortical dynamics during rapid movements or in reactive real-world scenarios. Many EEG analysis methods have been used to record electrocortical responses elicited by external stimuli. By studying changes in electrical potentials from the brain that are tied to an event of interest, such as auditory, visual, somatosensory, or vestibular cues, event-related potential (ERP) studies have uncovered changes in electrical brain activity during cognitive and motor behaviors (Kappenman and Luck, 2010). Event-related potentials represent phase-locked neural responses that can be measured during experimental manipulations (Gutberlet et al., 2009; Nidal and Malik, 2014) by repeating an event of interest (Galambos, 1992) to study the latency, morphology, and scalp topography of positive and negative voltage peaks and deflections. These analyses can be extended to study electrocortical changes in both time and frequency. During EEG analyses, the spectral power is used to study the distribution of signal power among frequencies (Sanei and Chambers, 2013) that are grouped into frequency bands based on functional roles and characteristics in the brain, including delta (1–3 Hz), theta (4–7 Hz), alpha (8–12 Hz), beta (15–30 Hz), and gamma bands (>30 Hz). Lower frequency bands indicate a subconscious state, while higher frequency reflects a more active and aroused state (Jensen et al., 2016). In order to track the temporal changes of the frequency spectrum, time-frequency EEG analyses were proposed. Event-related spectral perturbation (ERSP) analyses can show stimulus-induced, non-phase-locked brain activity over time (Tallon-Baudry et al., 1996; Rossi et al., 2014) and can provide insight into specific frequency bands that relate to functional brain processes (Rossi et al., 2014).

### Functional Connectivity Metrics

Beyond quantifying spatial, temporal, and spectral dynamics of electrical brain activity, the use of mobile EEG for studying the neural control of human locomotion can improve our understanding of functional interactions between brain structures and between brain and muscle during locomotor control. When significant temporal or spatial correlations are observed between neurophysiological processes, this phenomenon is often referred to as functional connectivity (Fingelkurts et al., 2005; Sakkalis, 2011). Coherence or correlation strength is considered directly proportional to the degree of functional connectivity between neuroanatomical structures when comparing electrophysiological signals (Thatcher et al., 1986; Im, 2018). To quantify signal interdependence without consideration for directional causation (Bullock et al., 1995; Kaplan et al., 1997), non-directed functional connectivity metrics such as correlation, coherence, mutual information, phase locking value, and pairwise phase consistency have been used (Bastos and Schoffelen, 2016). To quantify directed functional connectivity with consideration for causation, metrics such as cross-correlation, phase slope index, Granger causality, and transfer entropy, have been applied (Granger, 1969; Bastos and Schoffelen, 2016). These metrics can also be categorized based on considerations for signal amplitude or phase. Signal amplitude comparisons are conducted using correlation, mutual information, cross-correlation, Granger’s causality, partial directed coherence, transfer entropy, and dynamic causal modeling metrics (Im, 2018). Phase domain analyses include coherence, phase locking, pairwise phase consistency, and phase slope index (Im, 2018). Phase comparisons can also be assessed using Granger causality methods that include both parametric and non-parametric approaches (Geweke, 1982). Non-parametric Granger causality is calculated using autoregression and does not require model order to be determined (Bastos and Schoffelen, 2016). Parametric Granger causality is based on Fourier or wavelet-based methods, which require less data than non-parametric equivalents (Bastos and Schoffelen, 2016). For single-trial data and when model order is known, parametric Granger causality methods have shown greater sensitivity for quantifying neural functional connectivity compared to non-parametric Granger causality methods (Richter et al., 2015; Bastos and Schoffelen, 2016).

## Mobile EEG Data Processing Challenges and Solutions

### Physiological Artifacts and Solutions

Electrophysiological signals not limited to electrical brain activity are detectable from scalp EEG measurements. The heart rhythm, eye movements, and electrical muscle activity can influence each obscure electrocortical source activity and may also present contrasting signal characteristics that require specific noise removal strategies beyond independent component analysis. Cardiac activity is detectable from EEG measurements when the electrode is placed on or near a blood vessel (Goncharova et al., 2003). The repetitive low-frequency (~1.2 Hz) signal characteristics of the heart rhythm are distinguishable from cerebral activity, and removal can be assisted by relying on a reference waveform or an electrocardiogram (ECG; Jiang et al., 2019).

Ocular artifacts caused by eye blinks and saccades present large amplitude and low-frequency voltage fluctuations compared to EEG signals. Because of the different signal characteristics between brain activity and eye movements, electrooculographic (EOG) recordings from electrodes placed on the skin around the eye can help to parse EOG from EEG signals using ICA or regression methods (Jung et al., 1998; Li et al., 2006; Schlögl et al., 2007; Winkler et al., 2015). Alternative statistical decomposition methods that can be implemented in a sliding window to remove transient large amplitude artifacts have proven to be particularly effective. Artifact subspace reconstruction (ASR; Kothe and Jung, 2016) is a component-based artifact removal method that can be used to clean large-variance signal components based on thresholds compared to clean baseline data and subsequent reconstruction of EEG channel data. By pre-conditioning EEG channel data and removing eye movement and muscle artifacts ahead of ICA, it is possible to improve ICA decomposition quality (Chang et al., 2019).

Myoelectric artifacts can appear in EEG signals due to muscular contractions from the scalp, face, and neck. Electromyographic (EMG) recordings can present broad spectral distributions, including low and high frequencies (>200 Hz; Shackman et al., 2009; Urigüen and Garcia-Zapirain, 2015) but are usually more localized in higher frequency bands above 14 Hz (Narasimhan and Dutt, 1996). Conventional low pass filtering approaches can remove high-frequency signal content but may also eliminate electrocortical signals in beta (13–30 Hz) or gamma bands (>30 Hz) depending on the selected filter cutoff. To minimize the risk of undesirable signal loss, canonical correlation analysis (CCA) has been used to remove muscle artifacts from EEG data (De Clercq et al., 2006; Raghavendra and Dutt, 2011; Jiang et al., 2019). CCA measures the linear relationship between two datasets to derive signal components based on correlation or autocorrelation when derived from a single dataset, such as EEG channel recordings. Due to the high-frequency spectral characteristics of electrical muscle activity, canonical components with low autocorrelation tend to show high-frequency spectral content indicative of electrical muscle activity. Component removal or filtering can therefore help to eliminate myoelectric EEG signal contamination.

### Electromechanical Artifacts and Solutions

In addition to the mixture of electrophysiological signals captured by EEG recording electrodes, external noise sources can contaminate mobile EEG data. External artifacts can include alternating current power line noise, electromagnetic interference from electronic devices in the surrounding environment, and movement-related artifacts introduced by the movement of mobile EEG system components, such as cables and electrodes. Alternating current power line noise predominantly occurs at either 50 Hz or 60 Hz, depending on the country, and can largely be eliminated using a notch filter at the respective frequency band (Leske and Dalal, 2019). However, notch filtering can eliminate electrocortical target signals in gamma band (>30 Hz) depending on notch filter width. Alternative methods for power line noise removal have been implemented, such as Discrete Fourier Transform (DFT) filter (Oostenveld et al., 2011), frequency-domain regression (Bigdely-Shamlo et al., 2015), and spectrum interpolation (Leske and Dalal, 2019).

Inherent to the study of neural control of locomotion using mobile EEG, gait-related movement artifacts have posed non-trivial challenges to researchers. Small electrode motions on the scalp, cable sway (Symeonidou et al., 2018), and system component vibrations introduce signal contamination during each step of the gait cycle, causing voltage fluctuations in the EEG signal that exceed electrical brain activity. Signal fluctuations occur at the step frequency but can also extend into higher frequency bands at harmonics of the step cycle, with a non-uniform influence of noise among recording electrodes across the scalp (Kline et al., 2015; Snyder et al., 2015). Cable bundling and more effectively securing electrodes and system components to the participant can minimize motion artifact causes (Nathan and Contreras-Vidal, 2016), but it remains difficult to completely eliminate motion-induced noise through ICA decomposition methods alone. High pass filtering mobile EEG data provides a partial solution (Winkler et al., 2015), with a 1–2 Hz high pass filter improving subsequent ICA decomposition results, but a number of alternative signal processing solutions have been implemented in mobile EEG studies. Adaptive filtering (Kilicarslan et al., 2016), template regression (Gwin et al., 2010), and component-based statistical decomposition methods, including artifact subspace reconstruction (Chang et al., 2018, 2019), have been used to eliminate motion artifacts from mobile EEG data at relatively slow gait speeds (<1.0 m/s; Gwin et al., 2010; Wagner et al., 2012, 2016; Bradford et al., 2016, 2019; Oliveira et al., 2017a,b; Bradford et al., 2019), but gait speeds closer to, and in excess of, preferred human walking speed (1.4 m/s; Bohannon, 1997) have remained challenging and have required novel solutions.

### Automatic Preprocessing Toolboxes

Many EEG preprocessing procedures have been developed and incorporated into EEGLab toolboxes to provide standardized data analysis pipelines for improving rigor and reproducibility among mobile EEG studies (Bigdely-Shamlo et al., 2015; Gabard-Durnam et al., 2018; Pedroni et al., 2019). The PREP pipeline (Bigdely-Shamlo et al., 2015), AUTOMAGIC (Pedroni et al., 2019), and HAPPE (Harvard Automated Processing Pipeline for EEG) have each introduced functions for analyzing EEG data. These toolboxes include methods for identifying and removing unusually noisy channel data (findNoisyChannels; Bigdely-Shamlo et al., 2015; Pedroni et al., 2019), a multi-stage robust referencing scheme that eliminates noise from recorded EEG signals prior to computing a common average reference (Bigdely-Shamlo et al., 2015), alternating current powerline noise removal (cleanLineNoise and ZapLine; de Cheveigné, 2020), and artifact corrections for eye movements (Pedroni et al., 2019). Automatic open-source data processing toolboxes can improve consistency among mobile EEG analyses, but it remains important to ensure that mobile EEG hardware is configured to eliminate as many possible signal contaminating noise sources ahead of EEG data recording.

### Hardware-Assisted Solutions for Mobile EEG Motion Artifact Reduction

A common approach for mobile EEG artifact removal is to rely on simultaneously recorded reference signals from sources known to exist in the complex mixture of signals captured in EEG recordings (e.g., EOG, EMG, or ECG). Similar approaches using isolated motion and/or electrical noise recordings have been developed and applied. Using information about the participant’s head motions during gait is one possible solution for quantifying the causes of motion artifacts in mobile EEG. Optoelectronic motion capture, accelerometry, or inertial measurement units can be used for this purpose (Casson, 2019). Adopted from solutions to overcome significant signal contamination introduced by gradient artifacts during simultaneous MRI and EEG (Chowdhury et al., 2014), isolated noise recordings have recently been used to eliminate motion artifacts from mobile EEG during human locomotion using dual-layer EEG (Nordin et al., 2018). In this configuration, one layer of EEG electrodes measured a mixture of physiological signals and motion artifacts from the scalp, but the second layer of electrodes measured only electrical noise and motion artifacts from mechanically coupled but electrically isolated secondary electrodes. Noise-only electrodes were referenced to an overlaid conductive fabric cap that served as an artificial skin circuit but also more effectively secured the recording electrodes to the participant’s head. By conducting benchmark tests using a robotically controlled motion platform that reproduced human head motions during walking and an electronic head phantom device that generated ground truth artificial brain signals (Nordin et al., 2018; Richer et al., 2020), the ability of dual-layer mobile EEG for motion artifact removal was validated. These methods were subsequently applied during human treadmill locomotion at a range of gait speeds (Nordin et al., 2019a,b), including while navigating over unexpected obstacles on a treadmill belt (Nordin et al., 2019c).

## Mobile EEG for Studying The Neural Control of Locomotion

Recent mobile EEG studies have expanded our understanding of human supraspinal locomotor control, revealing electrocortical spectral power fluctuations tied to each step in the gait cycle (Wagner et al., 2012, 2016; Bradford et al., 2016, 2019; Luu et al., 2017a; Nordin et al., 2019a,c). Broadly distributed electrocortical network dynamics further show activations from the frontal cortex (Sipp et al., 2013; Bulea et al., 2015; Wagner et al., 2016), anterior cingulate cortex (Bulea et al., 2015; Bradford et al., 2016; Wagner et al., 2016; Luu et al., 2017a; Yokoyama et al., 2020), sensorimotor cortex (Wagner et al., 2012; Sipp et al., 2013; Bradford et al., 2016; Luu et al., 2017a; Nordin et al., 2019a; Yokoyama et al., 2020), auditory cortex (Wagner et al., 2016; Nordin et al., 2019a,b), supplementary motor area (Nordin et al., 2019c), premotor cortex (Nordin et al., 2019c), motor cortex (Wagner et al., 2012; Bulea et al., 2015), somatosensory cortex (Yokoyama et al., 2020), and the parietal cortex (Bulea et al., 2015; Bradford et al., 2016; Wagner et al., 2016; Luu et al., 2017a) during locomotor tasks ranging from steady treadmill gait to navigating over complex terrain or walking with robotic assistance. Knowledge gained from these studies provides the basis for understanding human electrocortical dynamics during balance and gait control that can be used for the development of assistive devices and neuroprostheses for rehabilitation, and to restore locomotor function for individuals with neurological disorders and disease.

### Locomotor Control

A growing body of evidence shows dynamic cortical activations during the initiation, maintenance, and modification of human gait (Choi and Bastian, 2007; Grillner et al., 2008; Wagner et al., 2012; Castermans et al., 2014; Bradford et al., 2016; Nordin et al., 2019c). During each step of the gait cycle, sensorimotor electrocortical spectral power modulations occur in alpha (8–12 Hz) and beta bands (13–30 Hz). Multiple studies have shown alpha and beta band spectral power increases during double support and decreases during limb swing of continuous gait (Gwin et al., 2010; Wagner et al., 2012; Bulea et al., 2015; Bradford et al., 2016, 2019; Oliveira et al., 2017b; Nordin et al., 2018, 2019a,b). Detectable changes in electrocortical spectral power have also been identified between uphill, downhill, and level treadmill gait (Bradford et al., 2016), walking with eyes closed compared to eyes open (Oliveira et al., 2017b), during transitions in gait speed (Wagner et al., 2016), and when navigating over complex terrain (Luu et al., 2017a). Compared to level and downhill walking (Bradford et al., 2016), during incline walking spectral power from the anterior cingulate cortex, sensorimotor cortex, and the posterior parietal cortex increased in theta band (4–7 Hz) and decreased in gamma band (>30 Hz). Walking with restricted vision induced desynchronization from theta to beta bands during the transition to single support for the somatosensory cortex (Oliveira et al., 2017b), suggesting that restricted vision increases sensory processing and integration compared to visually guided walking. Although changes in gait speed can be largely controlled subcortically, alpha and beta band sensorimotor electrocortical spectral power were shown to decrease at faster gait speeds (2.0 m/s) compared to slower walking (0.5 m/s; Luu et al., 2017a). While navigating complex overground terrain that included level ground, ramps, and stairs, participants showed reduced alpha and beta band spectral power from sensorimotor cortex during ramp and stair ascent compared to level-ground walking (Luu et al., 2017a). Beta and gamma-band spectral power also increased from the sensorimotor cortex during initial limb swing while ascending stairs. Collectively, these findings uncover a distributed network of cortical activity involved in movement control and sensory processing during gait, leaving considerable work to uncover dynamic interactions among brain structures and how these locomotor network dynamics differ among populations.

### Balance Control and Perturbation Responses

During bipedal gait, dynamic balance maintains upright posture by supporting body weight. The ability to maintain and recover from the loss of balance is critical to healthy gait function. The unexpected loss of balance due to external perturbations has been associated with electrocortical activations from primary sensory and motor cortices, supplementary motor area, premotor cortex, anterior cingulate cortex, prefrontal cortex, temporal cortex, parietal cortex, and visual cortex (Massion et al., 1999; Slobounov et al., 2009; Sipp et al., 2013; Marlin et al., 2014; Varghese et al., 2019). Loss of balance while walking on a balance beam has also shown greater theta band power and reduced beta power from the sensorimotor cortex compared to steady treadmill walking (Sipp et al., 2013). Divergent electrical brain dynamics also emerge during a loss of balance due to physical or visual perturbations (Peterson and Ferris, 2018). While walking on a balance beam, participants who experienced a physical pull at the waist, compared to a visual rotation of the environment using a virtual reality headset, showed increased spectral power from the sensorimotor cortex in the theta band and decreased beta power after perturbation onset (Peterson and Ferris, 2018). Compared to the loss of balance due to a physical pull at the waist, however, visual perturbations elicited more prominent responses from the parieto-occipital areas. During recovery from loss of balance during unexpected slips, compared to steady walking, spectral power from sensorimotor cortex similarly increased in the theta band and decreased in the alpha band, while alpha and beta band spectral power decreased from the parietal cortex (An et al., 2019). In response to unexpected obstacles that appeared on a treadmill belt during walking and running, event-related spectral power fluctuations from time-frequency analysis further identified spectral power increases from the premotor cortex, supplementary motor area, and the parietal cortex in the delta, theta, and alpha bands. The timing of electrocortical activation onset varied with locomotion speed, initiating two steps before stepping over the obstacle to enable foot placement planning around the obstacle (Nordin et al., 2019c). The ability to detect perturbation onset in advance of motor responses could provide bio signals for developing brain-machine interface technologies to properly assist in counteracting the loss of balance due to perturbations or changes in the environment during standing balance and gait.

### Mobile EEG for the Development of Neurotechnologies

Robotic-assistive devices are widely used for rehabilitation purposes to provide bodyweight support or to guide locomotor limb movements. To better understand the influence of robotic assistance on human locomotor control, researchers have studied changes in electrocortical spectral dynamics using mobile EEG. By comparing electrical brain activity during active treadmill gait to walking with assistive forces applied to the limbs or passive limb motions with bodyweight support, changes in sensorimotor processing have been uncovered. During robotically-assisted gait that provides bodyweight support and limb guidance, spectral power from premotor cortex and sensorimotor areas increased in alpha, beta, and gamma bands, compared to active treadmill walking (Wagner et al., 2012; Knaepen et al., 2014; Seeber et al., 2014). Recent mobile EEG studies that used a unilateral lower-limb exoskeleton to generate assistive joint torque outside the laboratory also showed hemispherical effects on parietooccipital regions in beta band compared to walking without robotic assistance (Li et al., 2018). As assistive robotic technologies for rehabilitation continue to develop, it becomes more important to better understand healthy human brain dynamics during locomotion. This knowledge not only informs how changes in electrocortical activity influence movement control, but also provides possible biomarkers for measuring adaptation to assistive devices or tracking rehabilitative progress and provides the mechanism for identifying electrocortical control signals that can be used for brain-machine interface technologies.

Brain-machine interfaces have shown increasingly promising applications for controlling output devices using direct communication with the human brain (Millán et al., 2004; Lebedev and Nicolelis, 2017; Tariq et al., 2018). Non-invasive EEG-based brain-machine interface systems can provide effective closed-loop strategies for deciphering user intentions while controlling physical or virtual machines, including multi-directional brain-actuated wheelchairs (Vanacker et al., 2007; Galán et al., 2008) or lower-limb exoskeletons (Noda et al., 2012; Contreras-Vidal and Grossman, 2013; Sczesny-Kaiser et al., 2015). Recent brain-computer interface demonstrations have allowed users to control a walking avatar in virtual reality using scalp EEG signals. In this application, alpha band spectral power from the posterior parietal cortex and inferior parietal lobe decreased along with increased gamma band spectral power from the anterior cingulate cortex, which has been attributed to error monitoring during walking (Luu et al., 2016, 2017b). Continued innovations using non-invasive EEG-based brain-machine interfaces can therefore advance current capabilities and lead to more intuitive assistive devices for rehabilitation, injury prevention, or human performance enhancement using the user’s own neurophysiological control signals.

## Discussion

In this review, we discussed mobile EEG technologies, including advancements in hardware and signal processing technologies for mobile applications. We identified different hardware strategies for improving signal recording quality and contemporary analytical methods for effectively extracting electrocortical source signals that can be localized to specific cortical structures with progressively better spatial resolution. We also identified the benefits of these fast timescale recordings for studying changes in voltages and spectral power that can be used to better understand how electrical brain activity changes during dynamic behaviors. Current state-of-the-art mobile EEG methods have led to considerable discoveries in the neural control of human locomotion, and through continued mobile hardware and signal processing innovations, new discoveries will continue to emerge that will enable studies in more realistic tasks and environments. Recent locomotion studies have revealed complex electrocortical dynamics that can be measured in time, space, and frequency. Effectively extracting and decoding these electrical brain signals will enable the development of robust, non-invasive brain-computer interface technologies for use in restoring, maintaining, and improving human gait.

Future improvements in mobile EEG technologies that enhance system usability will lead to more widespread adoption of these methods, including the use of compact, lightweight, and wireless system designs that can be entirely worn on the head and require reduced preparation time, but are also comfortable for the user to wear and remove to enable robust long-term recordings (Hairston et al., 2014; Izdebski et al., 2016; Oliveira et al., 2016; Bateson et al., 2017; Athavale and Krishnan, 2017). Analytical methods that cssan be realistically implemented in real-time for closed-loop applications will also enable the development of next-generation neurotechnologies. In addition to advancements in mobile EEG recording and analysis methods, unified approaches for simultaneously recording biosignals, such as eye gaze, electromyography, and biomechanical measures for quantifying whole-body human movement outside of conventional laboratory environments, will continue to expand the study of neural control of human locomotion into real-world scenarios.

## Author Contributions

SS and AN drafted and edited the manuscript. All authors contributed to the article and approved the submitted version.

## Conflict of Interest

The authors declare that the research was conducted in the absence of any commercial or financial relationships that could be construed as a potential conflict of interest.

## Publisher’s Note

All claims expressed in this article are solely those of the authors and do not necessarily represent those of their affiliated organizations, or those of the publisher, the editors and the reviewers. Any product that may be evaluated in this article, or claim that may be made by its manufacturer, is not guaranteed or endorsed by the publisher.
